# Spatiotemporal Analysis of the Proportion of Unimproved Drinking Water Sources in Rural Ethiopia: Evidence from Ethiopian Socioeconomic Surveys (2011 to 2019)

**DOI:** 10.1155/2022/2968756

**Published:** 2022-03-16

**Authors:** Abere Wondimu Kassie, Sintayehu Workineh Mengistu

**Affiliations:** Department of Statistics, College of Natural and Computational Science, Debre Berhan University, P. O. Box 445, Debre Berhan, Ethiopia

## Abstract

Currently, around 36% of the rural Ethiopian population is accessing drinking water from unimproved sources and it is unevenly distributed through time and geographic regions. Therefore, this study aimed to analyze the spatiotemporal patterns of unimproved drinking water sources and identify hotspot areas in rural Ethiopia. Ethiopian Socioeconomic Survey (ESS) data obtained from the Central Statistical Agency were used. It was conducted in four waves from 2011 to 2019. A two-stage probability sampling design was applied. The sample of enumeration areas and households were taken as the first and second stages of sampling, respectively. A total of 3912, 3775, 3698, and 3115 sample households with complete information on drinking water sources were taken in each wave of ESS data, respectively. Weighted proportions, autocorrelation (Moran's “I”) statistic, and hotspot analyses were applied to estimate the prevalence, test the presence of clustering, and identify vulnerable areas with unimproved drinking water sources. The STATA version 14, Excel, and ArcGIS 10.6 were used to manage and analyze data. The proportions of households with unimproved drinking water sources were 0.497, 0.385, 0.298, and 0.363 in consecutive waves of ESS data. The results also revealed the existence of geographical and temporal variations of access to drinking water from unimproved sources, and the most recent vulnerable (hotspot) areas in the borders of the West and East Gojjam zones in the western Amhara region, Zone one in southern Afar region, and Liben, Afder, Shebelle, Korahe, and Nobob zones in Somali region were identified. In conclusion, this study reveals significant geographic inequalities in the use of improved drinking water sources. This may be necessary for policies and coverage targeting the most vulnerable regions. The presented map and analytical approaches can provide a mechanism to monitor future reductions in inequality within countries by reflecting resource allocation priorities.

## 1. Introduction

According to the WHO and UNICEF Joint Monitoring Program (JMP), an unimproved source of drinking water is defined as a source of water that cannot adequately protect the source from external pollution, especially fecal pollution due to its structural nature [1]; this includes unprotected (dug) wells; unprotected springs, small tankers, or drums; and tanker water supply, surface water (river, dam, lake, pond, stream, canal, and irrigation canal), and bottled water (because the amount of water that households can obtain from this source may be limited, not the quality) [2]. However, improved drinking water sources are protected against outside contamination, notably fecal matter, through active interventions [3, 4], which includes tap water in residential plots or gardens, public faucets/standpipes, borehole/tube well, well digging and protection, protected spring, and rainwater collection.

An adequate, accessible, acceptable, and safe drinking water supply has to be available for various users. Following the recognition of the human right to safe drinking water [5], the United Nations Sustainable Development Goals (SDGs) were declared, including an objective to achieve equitable access and universal access to safe and affordable drinking water by 2030 (Goal 6 Target 6.1) [6, 7]. Hence, the access to and use of safe water can greatly contribute to health, productivity, and social development [8]. Unfortunately, in developing countries such as Ethiopia, the drinking quality of water is continuously being contaminated and is hazardous for human use due to high growth of population, expansion in industries, and throwing away of wastewater and chemical effluents into canals and other water sources [9].

Due to the lack of adequate drinking water services and the spread of disease from drinking, contaminated water is responsible for major outbreaks of diseases such as cholera and typhoid, including diarrheal disease and viral hepatitis A, cholera, dysentery, and Guinea worm disease [10]. For example, 85% of deaths from diarrhea and 1% of the global burden of disease are caused by unimproved drinking water and sanitation sources [11]. In the other instances, the impact of drinking water quality on infancy, where the risk of low birth weight in children from households with improved drinking water sources, is lower than in children with nonimproved water sources [12, 13].

According to a new report from UNICEF and WHO, billions of people in the world still have no access to water, sanitation services, and personal hygiene [14]; approximately 2.2 billion people in the world do not have safely managed drinking water services, 4.2 billion people do not have safely managed sanitation services, and 3 billion people lack basic hand washing facilities [14, 15]. When considering the problem in developing countries, the risk of contamination of drinking water is projected to increase significantly across tropical sub-Saharan Africa and developing countries in South-East Asia [3, 16]. What makes matters worse in these countries; geospatial datasets for drinking water sources often have necessarily limited resolution or inadequate spatial coverage [6].

In 2015, the JMP report stated that Ethiopia had achieved the Millennium Development Goals (MDGs), increasing the share of improved drinking water to 57% and the share of improved sanitation facilities to 28% [17]. Despite Ethiopia's remarkable progress, more than 48 million people still do not have access to improved water sources, and most sanitation facilities in the country do not have access to safe drinking water [17, 18]. Particularly, millions of people in rural areas in Ethiopia still do not get drinking water from an improved water source [8]. For this reason, Ethiopia is working to achieve target 6.1 of the Sustainable Development Goals (SDG) by 2030 to achieve universal and equitable access to safe and affordable drinking water for all [19]. Developing policy-related data and standardized methods for mapping water supply and sanitation services is needed to understand the inequality within and between countries and/or regions and help to determine the priority of resource allocation [11].

In addition, the distribution of drinking water varies depending on the residence and the type of source [19, 20]. In this regard, identifying highly vulnerable areas is essential in reducing the lack of improved water sources. Results of a few studies conducted in different settings of Ethiopia revealed that an unimproved source of drinking water was significantly clustered spatially [4, 21, 22]. However, the temporal pattern of unimproved drinking water sources was not incorporated in these studies and they used only one cross-sectional data; it may be unable to analyze the data in time and space domains [23]. In addition, taking drinking water source data aggregately may ignore urban-rural disparities. Therefore, this study aimed to analyze the spatiotemporal patterns of unimproved drinking water sources in rural Ethiopia using Ethiopian Socioeconomic Survey (ESS) data from 2011 to 2019.

## 2. Materials and Methods

### 2.1. Study Area

The study was conducted in Ethiopia. Ethiopia is located in the northeastern hemisphere, with latitude and longitude 9.1450° N and 40.4897° E, respectively (world population review). It is located in the Horn of Africa, with more than 112 million people (2019) having a rural share of 79%, the second most populous nation in Africa next to Nigeria, and the fastest growing economy in the region (the World Bank in Ethiopia 2020, Worldometers, accessed on July 22, 2021); however, it is still one of the poorest country with a per capital income of $850. Currently, Ethiopia has ten regional states. Furthermore, regions are subdivided into zonal administrations.

### 2.2. Study Data, Design, and Sampling

**Figure 1:**
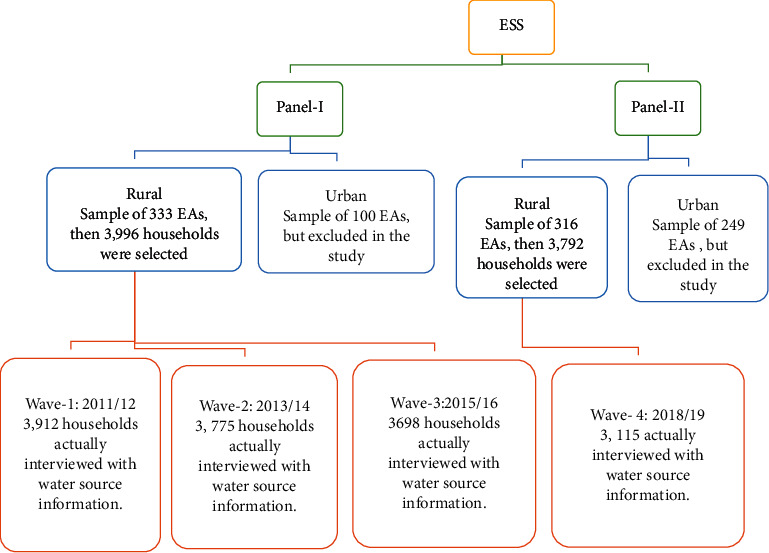
Sample and sampling technique of ESS data.

Data from the Ethiopian Socioeconomic Survey (ESS) provided by the Central Statistical Agency (CSA) were used. ESS aimed to collect panel data on a range of household and community characteristics related to agricultural activities in rural and urban areas [24–27]. However, the current study has taken data only from rural areas of ESS. The ESS was carried out in four rounds (waves) as presented in Figure 1. A two-stage probability sampling design was applied. In the first stage of sampling, enumeration areas (EAs) were taken. The second stage of sampling was the selection of households to be interviewed in each EA.

**Figure 2:**
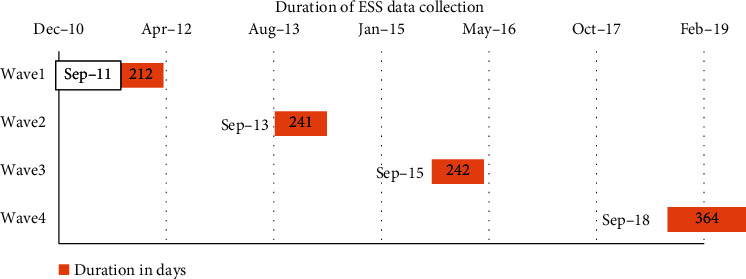
Gantt chart of ESS data collection schedule.

The four consecutive waves of ESS data collection periods are exhaustively shown in Figure 2. The first three waves were considered as panel-I [19] and conducted between 2011 and 2016. As shown in Figure 2, a one-year gap has been created due to the need for preparation time to refresh sample EAs and household lists since the fourth wave (2018/19) was under panel-II and taken as a baseline for future studies [20].

### 2.3. Spatial Autocorrelation Analysis

The Global Moran's “I” statistic was used to determine whether the unimproved drinking water source patterns are scattered, clustered, or randomly distributed in the study area [28]. Moran's “I” is a tool in spatial statistics, and it is used to measure spatial autocorrelation by taking the entire data set and producing a single output value in the range negative one and positive one. When Moran's “I” value is near to −1, 1, and 0, it represents unimproved water source spread, aggregation or clustered, and random distribution, respectively [29]. Moreover, when spatial autocorrelation is large and positive than the random expectation, it indicates the clustering of similar sources in the geographic space, while the significant negative spatial autocorrelation indicates that the neighboring sources of water are less similar than expected by chance and results in the existence of spatial patterns. The statistically significant Moran's “I” (*P* < 0.05) resulted in the rejection of the null hypothesis (random distribution of unimproved water sources) and indicated the existence of spatial autocorrelation [30].

### 2.4. Hotspot Analysis

In hotspot analysis, the Getis-Ord Gi *∗* statistics with its associated Z-score and *p* value at 95% confidence level were calculated to determine the statistically significant hotspots and clustering. When the z-score is between −1.96 and + 1.96, the *p* value is greater than 0.05 and could not reject the null hypothesis. It implied that the pattern exhibited could very likely be the result of random spatial process [31]. A statistical output with a high Gi *∗* indicates a “hotspot,” while a low Gi *∗* indicates a “cold spot” [32, 33].

Taking into account the complex sampling design of the survey such as cluster sampling and sample weights, the proportion of households not having improved drinking water sources was estimated. The STATA version 14, Excel, and ArcGIS 10.6 were used to manage and analyze data.

## 3. Results

### 3.1. Overall Rural Proportions of Improved and Unimproved Drinking Water Sources

The estimated proportions of unimproved drinking water sources were 0.497 (95% CI: 0.476–0.518), 0.385 (95% CI: 0.364–0.406), 0.298 (95% CI: 0.278–0.319), and 0.363 (95% CI: 0.340–0.388) for each of ESS data, respectively. In wave 1, the distribution of drinking water has not shown a significant difference between improved and unimproved sources, rather its proportion was almost fifty-fifty. The unimproved drinking water sources highly declined in the next three waves, especially at the third and second waves, while the improved proportions are higher than that of the unimproved ones (Table 1).

### 3.2. Proportions of Unimproved Drinking Water Sources among Regions and Their Temporal Patterns

In wave 1, Amhara, Oromia, and SNNP regions have higher proportions of unimproved drinking water sources with their estimated proportions, 0.490 (95% CI: 0.450–0.529), 0.539 (95% CI: 0.499–0.579), and 0.500 (95% CI: 0.457–0.543), respectively. In general, a significant decrement is observed among the proportions of unimproved drinking water sources in all regions other than regions considered as “others”. However, an exceptionally slight increment is shown at the fourth wave in Amhara, Oromia, SNNP, and “others” regions (Table 2).

### 3.3. Relationship of Households' Wealth Quintile Indices and Drinking Water Sources

**Figure 3:**
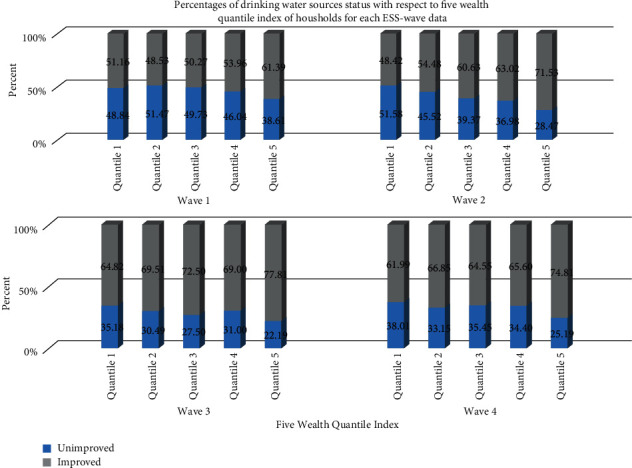
Improved and unimproved proportions of rural drinking water sources in terms of households' five wealth quantile index evidence from ESS data in four waves (2011/12, 2013/14, 2015/16, and 2018/19). The stacked bars show the percentages of improved and unimproved sources in each of the wealth indices.

Looking from the first to the fifth quintile (poorest to richest) of the four waves, a positive improvement in drinking water source is observed. It implied the percentage of unimproved drinking water sources is the lowest among the richest (upper wealth quintile) households and the highest percentage of unimproved drinking water sources is observed in the poorest (lower wealth quintile) households (Figure 3).

### 3.4. Spatial and Temporal Patterns

Each enumeration area (EA) in ESS data contained zero to twelve households who accessed their drinking water from unimproved sources. It was classified into quartiles (0 to 2, 3 to 5, 6 to 8, and 9 to 12) to enable clear visualization of EAs in a concentrated number of households that accessed an unimproved drinking water source.

**Figure 4:**
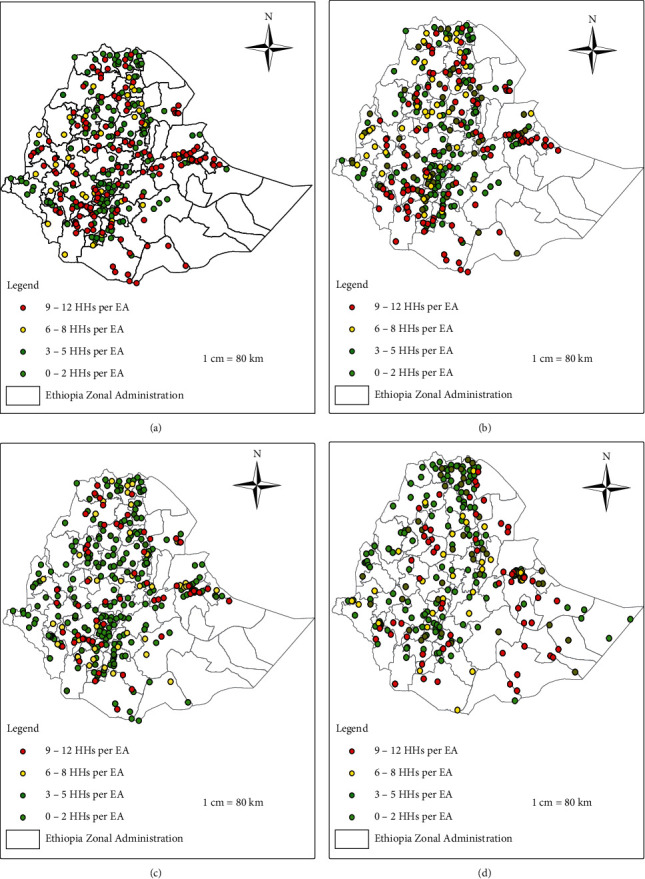
The spatial distribution of unimproved drinking water sources among households per EAs in rural Ethiopia evidence from ESS data in four waves, 2011/12, 2013/14, 2015/16, and 2018/19. In the figure, the raw data are divided into four quintiles (quartiles) at each wave based on the number of households whose drinking water sources have not improved in each EA. (a) Wave 1 (2011/12), (b) wave 2 (2013/14), (c) wave 3 (2015/16), and (d) wave 4 (2018/19).

During wave 1, EAs with higher burden of unimproved drinking water sources were largely distributed in the southwestern area of the SNNP, rural areas around Dire-Dawa and Harar, southern Afar, and western Amhara regions (Figure 4(a)) while northern Tigray, Gambella, central SNNP region, Oromo special zone, and South Gondar zone in the Amhara region were sparsely distributed. Southwestern and eastern Ethiopia were facing a high burden of unimproved drinking water sources whereas central and northwestern Ethiopia have the least burden second wave (Figure 4(b)). In wave 3, the clustering of unimproved drinking water sources was significantly diminished compared to the previous two waves, except in eastern and southwestern Ethiopia (Figure 4(c)). Compared to the former waves, a slightly higher burden was observed in wave 4, especially in eastern and northwestern Ethiopia and southwestern SNNP region (Figure 4(d)).

In overall, EAs with the lower burden of unimproved drinking water sources were located in central Ethiopia among all waves.

### 3.5. Analysis of Spatial Autocorrelations

**Figure 5:**
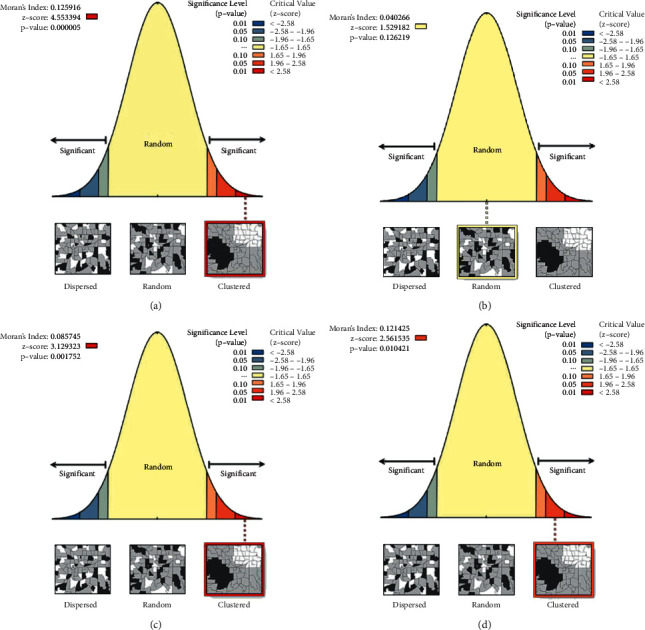
Test of spatial autocorrelation of rural unimproved drinking water sources in Ethiopia among households per EAs evidence from ESS data in four waves, 2011/12, 2013/14, 2015/16, and 2018/19. The broken red line on the right side of the figures indicates a high cluster pattern of unimproved drinking water sources at the first, third, and fourth waves of ESS data. Outputs were automatically generated in the upper left and upper right corners of the graph, and it explains that the probability of random clustering patterns is less than 1% except the second wave. (a) Autocorrelation of ESS-wave 1, (b) autocorrelation of ESS-wave 2, (c) autocorrelation of ESS-wave 3, and (d) autocorrelation of ESS-wave 4.

Figure 5 depicts whether drinking water sources are dispersed, random, or clustered spatially. Thus, the estimated autocorrelations were Moran's index = 0.125916, *Z*-score = 4.553394, and *p* value = 0.000005; Moran's index = 0.040266, Z-score = 1.529181, and *p* value = 0.126219; Moran's index = 0.085745, Z-score = 3.129323, and *p* value = 0.001752; and Moran's index = 0.121425, Z-score = 2.561535, and *p* value = 0.010421 for each wave of ESS data, respectively. Except for the fourth wave, all Moran's index values are positive, indicating a significant cluster of EAs containing households with unimproved drinking water sources at 5% level significance (Figure 5).

### 3.6. Hot Spot Analysis

**Figure 6:**
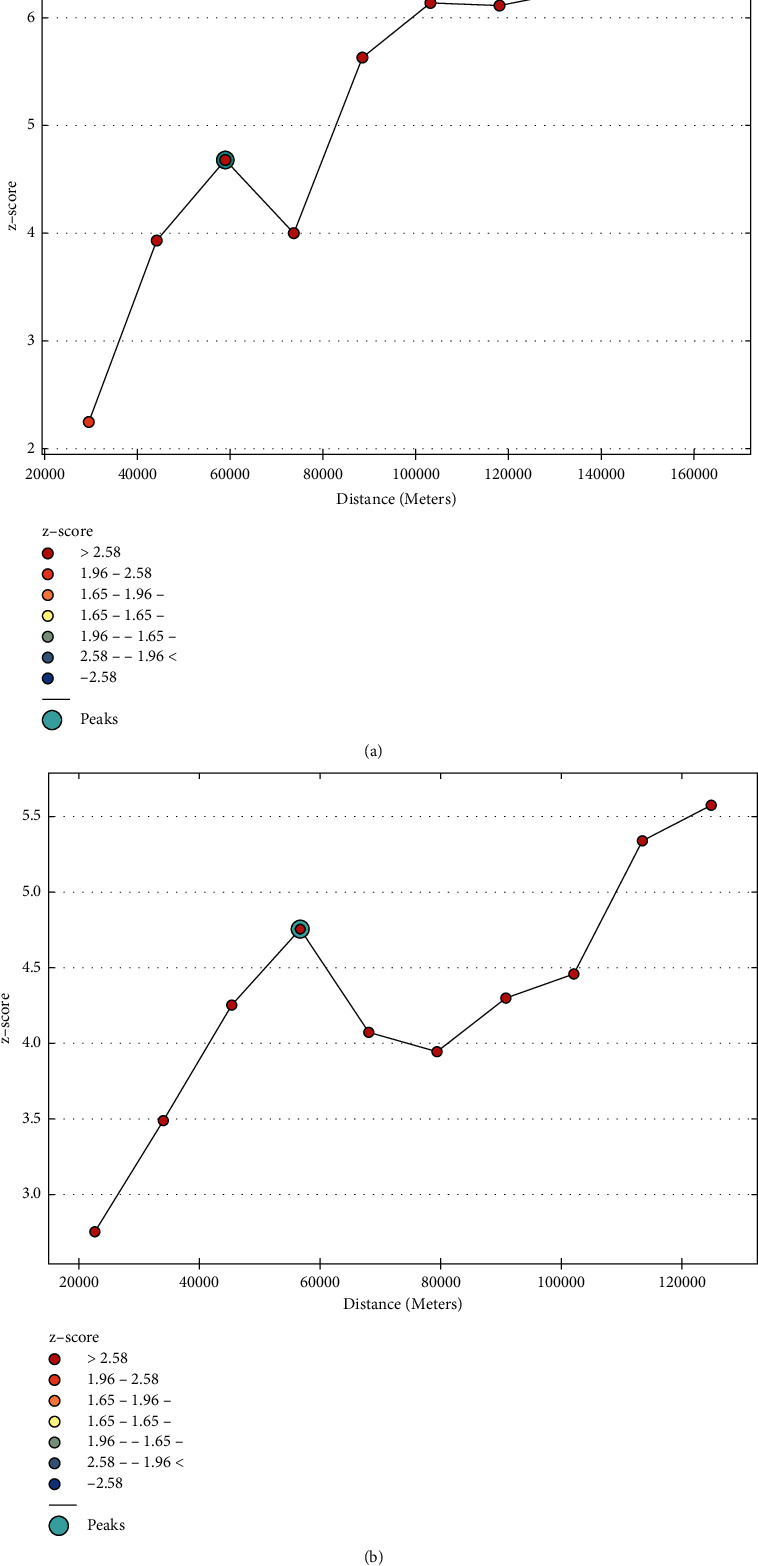
Incremental autocorrelations of ESS data for the first three waves (2011/12, 2013/14, and 2015/16) (a) and for the fourth wave (b). Graphs ‘a' and' b' have one and two peaks (maximum values), respectively, and used as the threshold distance (distance band) in the hotspot analysis. (a) Incremental autocorrelation by distance band for ESS data, wave 1,2,3. (b) Incremental autocorrelation by distance band for ESS data, wave 4.

An incremental autocorrelation was estimated using “distance band” to identify the most vulnerable areas with unimproved drinking water sources as shown in Figure 6.

**Figure 7:**
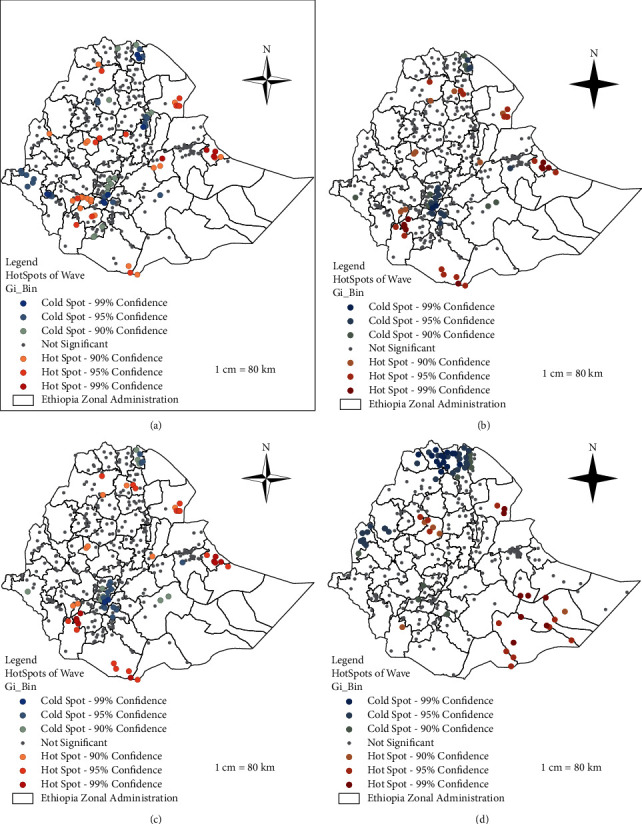
Hotspot area identification of unimproved drinking water sources in rural Ethiopia, evidenced from ESS conducted in four waves (2011/12, 2013/14, 2015/16, and 2018/19). The most dark red colors show significant clusters (highly vulnerable or hot spot areas) of unimproved water sources at 1% level of significance, the next dark red and bright red colors also show significant clusters of unimproved water source areas at 5% and 10% level of significance, respectively, whereas the most dark blue, the next dark blue, and bright blue colors show significant nonvulnerable (cold spot) areas at 1%, 5%, and 10% level of significances, respectively. (a) Hot spot analysis of ESS-wave 1, (b) hot spot analysis of ESS-wave 2, (c) hot spot analysis of ESS-wave 3, and (d) hot spot analysis of ESS-wave 4.

The Gi_Bin field classifies the data in the range of negative 3 (99% confidence for cold spots) to positive 3 (99% confidence for hot spots), being 0 not significant. Therefore, areas within the positive confidence level of 90% to 99% were indicated as hotspot areas for households that did not have access to improved drinking water sources (Figure 7).

## 4. Discussion

This study aimed to analyze the spatial and temporal patterns of unimproved drinking water sources in rural Ethiopia using Ethiopian Socioeconomic Survey (ESS) from 2011/12 to 2018/19. From the results of the study, about 64% of the rural population of Ethiopia has access to safe drinking water. The remaining 36% of the rural population is currently forced to use unsafe drinking water.

At the national level but rural only, the proportions of drinking water from unimproved sources were varied across the five quintile wealth indices of households. The result further revealed that the proportion improved source increases as the household wealth quintile index increases, especially in the third wave of ESS data, and it was consistent with a study [2]. Another study [4] also confirmed the current study as the proportions of households with lower unimproved water coverage were related to the latter two (fourth and fifth) quintiles.

Our study show, consistent with findings [11, 21], that the proportions of unimproved drinking water sources were found to vary geographically over the study period. For instance, it was found to be the lowest in the Tigray region followed by SNNP, Amhara, and Oromia regions during the first three waves of ESS data (Table 2) and it was consistent to [26]. Variations were seen not only between administrative areas but also within regions and zones. Understanding geographic disparities in improved drinking water supply can provide insights into resource allocation and prevent future water-related problems.

A study [22] revealed an apparent clustering trend of unimproved water coverage between regions. Similarly, in the current study, a spatial clustering was found to be significant in some areas. Moreover, the most vulnerable areas forced to drink water from unimproved sources were also identified. The hotspot analysis detected the variations of its source systematically and powerfully confirmed by Moran's “I” statistics.

According to our results, clusters in the North Gondar zone of the Amhara region, Zone one in the Afar region, Fafan zone in the Somali region, and areas shared (in Kaffa, Dawro, South Omo zones and Konta special woreda) in the SNNP region were identified as hotspots of the unimproved drinking water sources throughout the first three waves of ESS data. In the Tigray region (western Tigray zone), Oromia region (Horo Gudru and southeastern Borena zones), Amhara region (eastern Gojjam and northern Shewa zones), Benishangul- Gumuz region, and Somali region (west Liben zone) were also identified as hotspots in the first wave (2011/12).

Consistent to another study [4], Jimma and Hararge zone in Oromia, Sitti zone in Somali, and rural Dire-Dawa newly emerged as hotspots, whereas western Tigray, and Liben zone in Somali were reversed to risk areas of unimproved potable sources during the third wave (2015/16). During the same period, Horo Gudru, South Gondar, Wag Hemera, and South Tigray zones showed an improvement in terms of drinking water sources. In line with the result of study conducted by [34], in the fourth wave (2018/19), hotspots shifted to the border of west and East Gojjam zones in the Amhara region, Zone one in the south Afar region, and Liben, Afder, Shebelle, Korahe, and Nobob zones in the Somali region.

## 5. Conclusion

This study revealed that the proportions of unimproved drinking water sources in rural Ethiopia has shown a significant reduction from 0.497 to 0.363 across survey periods, 2011/12 to 2018/19; however, it still indicates a high prevalence. The study also revealed significant geographic disparities in the use of improved drinking water sources. This may be necessary for policies and coverage targeting the most vulnerable areas. The presented maps and analytical approaches can provide a mechanism to monitor future reductions in inequality within rural Ethiopia by reflecting resource allocation priorities.

## Figures and Tables

**Table 1:** The proportions of improved and unimproved drinking water sources in rural Ethiopia using ESS data conducted between 2011/12 and 2018/19.

Wave	Status	Proportion	Sd. error	95% CI
Wave 1: 2011/12	Unimproved	0.497	0.011	0.476–0.518
	Improved	0.503	0.011	0.482–0.524

Wave 2: 2013/14	Unimproved	0.385	0.011	0.364–0.406
	Improved	0.615	0.011	0.594–0.636

Wave 3: 2015/16	Unimproved	0.298	0.010	0.278–0.319
	Improved	0.702	0.010	0.681–0.722

Wave 4: 2018/19	Unimproved	0.363	0.012	0.340–0.388
	Improved	0.637	0.012	0.612–0.660

**Table 2:** Proportions of unimproved drinking water sources among regions and its temporal patterns in rural Ethiopia using ESS data conducted between 2011/12 and 2018/19.

Region	Wave	Proportion	Sd. error	95% CI
Tigray	1	0.326	0.027	0.275–0.381
	2	0.368	0.029	0.314–0.426
	3	0.287	0.027	0.238–0.342
	4	0.120	0.017	0.091–0.156

Amhara	1	0.490	0.020	0.450–0.529
	2	0.437	0.020	0.398–0.477
	3	0.331	0.019	0.295–0.369
	4	0.385	0.023	0.341–0.431

Oromia	1	0.539	0.021	0.499–0.579
	2	0.341	0.019	0.304–0.380
	3	0.321	0.020	0.283–0.361
	4	0.351	0.023	0.307–0.398

SNNP	1	0.500	0.022	0.457–0.543
	2	0.378	0.021	0.337–0.420
	3	0.225	0.017	0.193–0.260
	4	0.393	0.024	0.347–0.442

Others^*a*^	1	0.474	0.025	0.424–0.523
	2	0.474	0.026	0.424–0.524
	3	0.259	0.021	0.219–0.303

Afar	4	0.525	0.037	0.451–0.597
Somali	4	0.588	0.027	0.534–0.640
Benishangul-Gumuz	4	0.105	0.024	0.660–0.162
Gambella	4	0.276	0.330	0.216–0.346
Harari	4	0.260	0.035	0.198–0.334
Dire-Dawa(rural)	4	0.283	0.044	0.204–0.378

^*a*^Afar, Somali, Benishangul-Gumuz, Gambella, Harari, and Dire-Dawa.

## Data Availability

The data used to support the results of this study are available on request from the corresponding author.

## Abbreviations

CI: Confidence interval
CSA: Central Statistical Agency
DHS: Demographic and Health Survey
EAs: Enumeration areas
ESS: Ethiopian Socioeconomic Survey
JMP: Joint Monitoring Program
HHs: Households
MDGs: Millennium Development Goals
SNNP: Southern Nations, Nationalities, and Peoples' Region
UN: United Nations
UNICEF: United Nations International Children's Fund
WHO: World Health Organization.
